# Exploring potential salivary biomarkers for dental caries: a systematic review

**DOI:** 10.4317/medoral.27329

**Published:** 2025-08-16

**Authors:** Mónica López-Galindo, William Atashkadeh

**Affiliations:** 1 Associate Professor. PhD. Dentistry Department. Faculty of Health Sciences. European University of Valencia, Spain; 2 Dentist graduated from European University of Valencia, Spain

## Abstract

**Background:**

Dental caries remains one of the most widespread non-communicable diseases. Saliva is crucial for maintaining oral health as it shields teeth from demineralization and promotes the remineralization of enamel. Although ongoing studies are investigating the relationship between various salivary proteins and dental caries, consensus in existing literature has not yet been established. This study aims to provide additional insights into the current research of salivary protein biomarkers association with dental caries.

**Material and Methods:**

This systematic review analyzed literature published between January 2013 to December 2023, retrieved from PubMed, Scopus, and Web of Science.

**Results:**

The review included 21 observational studies (2 cohort, 2 case-control, and 17 cross-sectional) involving over 2,000 participants, examining 18 different proteins. There was considerable variability in the types of salivary markers studied. Among the participants, 54% were diagnosed as caries-active (CA), while 45.9% were caries-free (CF), with ages ranging from 6 to 89 years. The Newcastle-Ottawa Scale indicated that the risk of bias was low in 10 studies, intermediate in 10, and high in 1.

**Conclusions:**

Eighteen studies found significant differences in protein expression between CA and CF subjects, underscoring the potential of using salivary biomarkers for non-invasive diagnose assessment. However, larger and greater designed studies are needed to establish their clinical value. Besides, divergent results from proteomic studies on biomarkers may be due to variations in genetics, diet, oral hygiene, age and other factors of the subjects, which could affect the reliability of saliva biomarkers in caries screening and detection. The significant heterogeneity among studies made conducting a proper meta-analysis infeasible.

** Key words:**Biomarkers, dental caries, protein, proteomics, saliva, salivary biomarkers, biomarkers, systematic reviews.

## Introduction

Dental caries stands as one of the most prevalent non-communicable diseases (1) and according to Global Burden of Disease study 2019, untreated caries affects over 2 billion people worldwide (2). Although caries has significantly decreased globally and even though caries is largely prevenTable, it still remains the most prevalent chronic disease in both children and adults (3). Characterized by the breakdown of tooth structure due to the acidic byproducts produced by cariogenic bacteria. The multifactorial disease process of caries is significantly influenced by lifestyle choices and host factors, with saliva playing a crucial role (4).

Being able to detect and diagnose dental caries at an early stage, play a crucial role in preventing irreversible tooth structure (5). The functions of saliva extend beyond the mere processing of food for digestion. Saliva encompasses a diverse array of proteins, crucial for regulating the immune defense and the maintenance of mucosal tissue. Additionally, salivary proteins play a central role in protecting the dental structures and oral integrity (6).

As a result, being able to measure alterations in the quantity and composition of saliva could have the potential to serve as a valuable tool for the detection and monitoring of caries. Consequently, more attention has been growing on this topic, (7). According to the National Institute of Health (NIH), a biomarker is defined as “A characteristic that can be objectively measured and evaluated as an indicator that reflects normal biological process, pathogenic process or pharmacological process to a therapeutic intervention” (8). Examining the role of salivary biomarkers in the pathophysiology of caries, has led to a noTable increase in relevant studies over the past decade (9,10). However, this surge has led to controversies and findings that are challenging to reproduce or conflicting, primarily due to variations in methodological designs, statistical analyses, and the interpretation of study results (11). Therefore, the aim of this review was to explore the connection between caries and salivary proteins, comparing those with and without caries experience, and investigating whether salivary proteins could be identified as reliable biomarkers for caries.

## Material and Methods

The present systematic review was carried out following the statement of the PRISMA Guide (Preferred Reporting Items for Systematic reviews and MetaAnalyses) (12).

-Protocol and focus question.

The Medline-PubMed database (United States National Library of Medicine), Web of Science and Scopus were used to search for indexed articles which evaluated different salivary biomarkers/proteins and assessed concentration levels of various potential biomarkers/proteins in patients with either caries or without caries experience or patient considered caries free or caries active, published from January 2013 until December 2023 to answer the following question: In healthy individuals, does the analysis of salivary biomarkers have the potential to enhance the detection and diagnosis of dental caries?

This study question was set according to the PICO structured question. The question format was established as follows:

P (population): Healthy Individuals with no current disease.

I (intervention): Salivary biomarkers/proteins for caries presence or absence.

C (comparison): Caries active vs caries free subjects.

O (outcome): Levels of salivary biomarkers/proteins concentrations between subjects that are caries free vs caries active.

-Selection criteria.

Inclusion criteria.

Type of study: Publication in English, more than > 20 participants of patients, publications in the last 10 years, *in vivo* studies, cohort studies, cross-sectional and case-control studies.

Type of Population: Healthy individuals not under any medication older than 6 years old.

Type of intervention: Assessing salivary proteins in dental caries free vs caries active individuals.

Type of results: Articles that assess concentration levels of different salivary biomarkers/protein in caries or non-caries individuals.

Exclusion criteria.

Exclusion criteria were as follows: Assessing early childhood caries, reviews, reports, unpublished articles, *in vitro* studies, books and in animals.

- Search strategy

An electronic search was carried out in the three databases of PubMed, Scopus and Web of Science with the purpose of identifying studies related to salivary biomarkers/proteins and caries. The search strategy was carried out with the help of the PICO question. The search words used were: “adult”, “child”, “adolescent”, “biomarker”, “proteins”, “protein”, “peptides”, “indicator”, “marker”, “markers”, “caries”, “dental caries susceptibility”, “tooth decay”, ”saliva”, “unstimulated saliva”, “stimulated saliva” The search words were connected using the operators “AND” and “OR”. In all 3 databases the same filters were applied, which were published articles between 2013 and 2023, with English language. In the database Scopus, the articles were limited to filter “subject area dentistry”.

- Selection process

The studies selection process was carried out in two stages. Firstly, the duplicates were removed with Mendeley and then the first stage of screening followed. The title and the abstract of each article were read, and the inclusion and exclusion criteria were applied. Then the second stage of screening followed which the entire article was read, and the inclusion and exclusion criteria were applied. Articles which did not fit the inclusion criteria were discarded and collected in a Table with their author's name, publication years, title of article and reason for exclusion. The remaining articles were included in the systematic review. Every step was filled in the PRISMA 2020 flow diagram.

- Data Collection

The selection of the studies was carried out by two reviewers (WA, ML). A selection process was carried out. The following data were extracted from the included studies: author’s surname and year of publication, age of subjects, gender, subjects with caries and without caries, caries index, type of saliva collected, saliva amount (ml), salivary protein assessed, levels of salivary protein, method for assessing concentration of salivary protein (Table 1).

- Risk of bias tool and quality assessment

To measure the quality of non-randomized observational studies, the Newcastle-Ottawa Scale was used (13); “low risk of bias” was considered in the case of a ≥ 7-9, “intermediate” for 5-6 points and “high risk of bias” in the case of of ≤ 4. The NewcastleOttawa Scale (NOS) is a validated, easy to use scale of 8 items in three domains, selection, comparability, and outcome. The NOS was used to assess the quality of the included studies (Table 2). Studies are graded one point each for all items except comparability which has the potential to score up to two points, with the maximum possible score being nine.

- Data synthesis

The extracted data were divided into qualitative and quantitative variables. The qualitative variables were the following: authors name, country, saliva sample collection method, protein assessed, caries index used, methods used to assess the salivary biomarker. Whereas the quantitative variables were the following: sample size, age, sex, differences, subjects with caries and subjects without caries. Besides we will extract the salivary concentrations of each protein and the mean value levels of the proteins being assessed in both groups of caries and non-caries subjects. Furthermore, we will evaluate if there is a significance in the concentration levels of the biomarkers between the caries free and caries active subjects in the included studies (Table 3). Both qualitative and quantitative variables will be discussed in the synthesis section.

## Results

- Study selection

A total of 687 articles were initially identified as potentially relevant records from the databases searched. Following duplication, 121 duplicate sources were removed, leaving (566) unique sources. Subsequently, the titles and abstracts of these sources were screened, resulting in the exclusion of 531 articles that did not align with the study objective. The remaining 35 records underwent full-text screening based on predefined eligibility criteria. Of these, 14 studies were deemed ineligible and excluded. Finally, 21 records m*et al*l inclusion criteria and were considered for inclusion in the study. Further details of the search and selection outcomes are provided in Table 1.

As presented in Table 3, several salivary proteins were evaluated in the studies included of this review. Encompassing mucin, proline-rich proteins (PRP), cathelicidin (LL-37), immunoglobulin (IgA), statherin, total protein, superoxide dismutase (SOD), proteinase 3 (PR3), α-amylase, carbonic anhydrase (CAIV), matrix metalloproteinases (MMPs), tumor necrosis factor (TNF-α), interleukin (IL), beta-defensin (HBD), sCD-14 and vastatins were all assessed. Out of the 21 studies, a statistically significant increase in the salivary levels of IgA (14-17), α-amylase (17), proteinase-3 (18), Mucin (19) Statherin (20), was found in the caries-free subjects.

While nine of the studies of salivary levels of YKL-40 (21) APRP-½ (22), Superoxide dismutase (SOD) (23), MMP-8 (24), sCD-14 (25), s-IgA (26) observed to be significantly increased in caries-active subjects. Whereas in three articles (27,28) no statistically significant association was observed between the salivary levels of among CF and CA individuals. Regarding total protein among CF and CA subjects, three included studies (15,24,29) found a statistically significant increase in CA subjects whereas (27) found no statistical difference.

-Risk of Bias Assessment of Included Studies

The included studies were classified into high, moderate, and low risk of bias (RoB) with the Newcastle-Ottawa scale for observational studies. Of the 21 included publications, 10 were categorized as low, 10 as medium, and 1 as high. The Risk of bias as evaluated by the Newcastle-Ottawa scale varied significantly across the included articles, ranging between 4/9 and 8/9 stars. (Table 2).

## Discussion

Due to significant heterogeneity among the included studies, no meta-analysis could be performed. The most prevalent biomarker studied in this review was salivary immunoglobulin A (IgA) biomarker. The findings of this review revealed that salivary IgA levels were upregulated among CF individuals in five (14-17) except for the one conducted by Khan *et al*., (26) which reported significantly higher levels in CA subjects. Similarly, in accordance with this review, the meta-analysis by Wu *et al* 2020 (30) found salivary levels of IgA to be significantly reduced in CA. Besides earlier published literature (30,31), associated caries susceptibility with decrease in salivary IgA. Now the upregulation in CF could be explained by the significant protective role of immunoglobulins in oral health. IgA is the primary immunoglobulin found in human secretions and mucosal surfaces, providing a critical defense against oral diseases.

Four of the included studies measured the overall total protein levels between CF and CA subjects. All four studies related to total protein concentrations in this review found that indeed higher total concentrations levels were associated with increased caries incidence, Razi *et al*., (15) Hedenbjörck-Lager *et al*. (24), Vasudevan *et al*. (27), Pyati *et al*. (29) however Vasudevan *et al*. (27) could not establish any statistical significance between the CF and CA subjects. In accordance with this review, the systematic review and meta-analysis conducted by Silveira *et al*. (33) found that CF individuals had lower concentrations of total protein compared to CA subjects. Literature reports that total protein concentration increases with the expansion of cavitated carious lesions due to the degradation of dentinal matrix by MMPs. As a matter of fact, MMP activity was significantly upregulated in CA subjects in the included study (24), which strengthens this theory and positive correlation previously noted.

-Significance of the study 

Dental caries involves a complex interplay of factors (1), making it difficult to identify a single component for predicting or diagnosing disease progression or severity. Scholars highlight the critical role of saliva as the primary natural defense mechanism for oral health, protecting tooth surfaces through multiple functions such as mechanical rinsing, buffering capacity, and antimicrobial activity. Traditional methods such as visual-tactile exams, radiographs and dyes are common in dental caries detection (13). However, saliva sampling, being noninvasive and easily accessible, offers advantages

for biochemical testing compared to blood and conventional methods. Until now, saliva tests currently focus on properties like viscosity, pH, and flow rate to determine patient caries risk. Therefore, this systematic review aimed to identify potential salivary biomarkers that could be useful for detection and diagnosis of caries.

Limitations of the study

It's crucial to acknowledge the limitations of this systematic review. Primarily, all of the studies included in the review were observational studies (2 cohort, 2 case-control, 17 cross-sectional) designs. Consequently, there is a need for additional research, particularly longitudinal cohort studies with adequate follow-up periods. Future studies investigating salivary biomarkers should prioritize the implementation of standardized protocols for saliva sample collection, treatment procedures, and analytical methods. Additionally, there is a need for uniformity in data analysis protocols. Such studies will help to refine and organize our understanding of this research area before we can confidently consider salivary proteins as reliable biomarkers for caries detection. Besides, Saliva's composition and biological attributes are subjected to multiple factors, such as age, saliva flow rate, type of stimulus, gland type, diet, hydration, and circadian rhythm (25). Ensuring adequate control and adjustment of these variables is of great importance to minimize potential sources of variability and bias in analyses of biomarkers.

## Conclusions

Based on the results of this systematic review, 18 out of 21 studies identified a statistically significant difference between caries-active (CA) and caries-free (CF) subjects. As seen in this review, differences existed in protein expression between individuals with and without caries which highlights the potential of using saliva for non-invasive assessment of caries risk. However, determining whether salivary biomarkers have clinical value, larger studies and better study designs are needed in the future. Besides, the many divergent results from saliva proteomic studies can be attributed to differences in genetics, food habits, diet variation, oral hygiene routines, age and more. These variables may also obstruct a robust and reliable use of saliva proteomics for

screening and detection of caries in the future. Due to significant heterogeneity among

the studies, conducting a meta-analysis was not feasible.

## Figures and Tables

**Table 1:** General sample characteristics of the included studies.

Author & country	Age(years)	Sample size (male : female)	Subjects caries active: caries free	Caries index (low, high)	Saliva & amount	Method for assessing protein
Duruk et al.,(2022) Turkey (98)	6-15	80:(38:42)	60:20	Dmft/DMFT Dmfs/DMFS ICDAS Pufa/PUFA	2 mL Unstimulated saliva	ELISA
Hataysal et al.,(2022) Turkey(99)	18-54	165(75:90)	115:50	DMFT	Stimulated saliva	ELISA
Khan et al,.(2021) Pakistan (21)	20-50	43:N/A	33:10	ICDAS	2 mL, Unstimulated saliva	ELISA
Ahmad et al.,(2021) India (88)	8-12	100:(48:52)	50:50	DMFT (0,≥5)	2-3 mL whole unstimulated saliva	ELISA VITROS AMYL SLIDES
Vasudevan et al.,(2021) India(94)	6-12	60	30:30	DMFT (0,≥5)	6ml Stimulated saliva	ELISA
Al-Ali et al.,(2021) Iraq (104)	6-7	88(44:44)	44:44	Dmfs/ICDAS(0,>=4)	5mL, unstimulated saliva	ELISA
Angarita-Diaz et al.,(2021) Colombia (108)	6-12	33:(14:19)	21:12	ICDAS	2 mL, Unstimulated saliva	ELISA
Razi et al.,(2020) India (95)	12-15	40:(20:20)	20:20	DMFS(0,≥10)	5 mL, Unstimulated saliva	Single radial immunodiffusion
Al-ani (2020) USA (107)	18-70	60:(27:33)	28:32	CAMBRA	2 mL, Unstimulated saliva	ELISA
Karpinska et al.,(2019) Poland (71)	25-40	45(22:23)	26:19	DMFT(0,>13.9)	2 mL, Unstimulated saliva	ELISA
Pyati et al.,(2018) India (78)	6-12	100:(57:43)	50:50	DMFS/dfs(0,≥5)	0.5 mL Unstimulated saliva	Biuret method
Nazemisalman et al.,(2018) Iran (102)	3-18	79(30:49)	45:34	Visual detection	5 mL, Unstimulated saliva	ELISA
Karpinska et al.,(2018) Poland(103)	25-42	48:(30:18)	28:20	DMFT(<5, >13.9(	N/A, unstimulated saliva	ELISA
Sitaru et al., (2017) Romania (87)	10-14	128:(63:65)	84:44	DMFT (0-2,3-5)	10 mL Unstimualted saliva	Spectrophotometer
Prester et al.,(2017) Croatia (72)	20-40	55:(20:35)	30:25	DMFT	Stimulated & unstimulated saliva	ELISA
Yang et al., (2015) China (105)	6	128:(63:65)	82:46	DMFT(0-4,5-15)	5mL, unstimulated saliva	ELISA
HedenbjörckLager et al.,(2015) Sweden (106)	18-89	451(219:232)	145:306	DMFT	Stimulated saliva	ELISA Bradford Immunofluorometric
Cogulu et al.,(2015) Turkey (100)	6-12	108:(53:55)	74:34	DMFT(0,1-4,>4)	Unstimulated whole saliva	ELISA
Hegde et al.,(2014) India (96)	25-50	80: N/A	60:20	DMFT(0,>10)	5 mL, Unstimulated saliva	Beauchamp and Fridovich method
Golpasand Hagh et al., (2013) Iran (101)	19-45	70:(44:26)	30:40	DMFT (0,≥5)	N/A Unstimulted saliva	ELISA
Pal et al., (2013) India (97)	6-14	45:N/A	30:15	DMFT/(0,1-4. ≥5)	4 mL, Unstimulated saliva	ELISA

**Table 2:** Risk of bias assessment (Newcastle Ottawa Scale).

Study	Selection	Comparability	Exposure	Total score
Duruk et al.,(2022) Turkey (98)	★★★	★★	★★	7
Hataysal et al.,(2022) Turkey (99)	★★★	★★	★★	7
Khan et al,.(2021) Pakistan (21)	★★★	★	★★	6
Ahmad et al.,(2021) India (88)	★★★★	★	★★	7
Vasudevan et al.,(2021) India (94)	★★★	★	★★	6
Al-Ali et al.,(2021) Iraq (104)	★★★★	★★	★★	8
Angarita-Diaz et al.,(2021) Colombia (108)	★★★	★	★	5
Razi et al.,(2020) India (95)	★★★★	★★	★★	8
Al-ani (2020) USA (107)	★★	★★	★★	6
Karpinska et al.,(2019) Poland (71)	★★★	★★	★★	7
Pyati et al.,(2018) India (78)	★★★★	★	★★	7
Nazemisalman et al.,(2018) Iran (102)	★★★	★★	★★	7
Karpinska et al.,(2018) Poland (103)	★★	★	★	4
Sitaru et al., (2017) Romania (87)	★★★	★	★★	6
Prester et al.,(2017) Croatia (72)	★★	★	★★	5
Yang et al., (2015) China (105)	★★	★	★	5
HedenbjörckLager et al.,(2015) Sweden (106)	★★★	★★	★★	7
Cogulu et al.,(2015) Turkey (100)	★★★	★	★★	6
Hegde et al.,(2014) India (96)	★★★★	★	★	6
Golpasand Hagh et al., (2013) Iran (101)	★★★★	★	★★	7
Pal et al., (2013) India (97)	★★★	★	★★	6

Measurement of the risk of bias of non-randomized observational studies with the Newcastle-Ottawa scale- observational studies with a non-randomized control group.

**Table 3:** Biomarker concentration levels between caries-active and caries-free subjects.

Author	Salivary proteins	mean ± SD Concentration protein in caries-active (case)	mean ± SD Concentration protein in caries-free (control)	P value of biomarker
Duruk et al.,(2022) Turkey (98)	YKL-40	68.17±18.27	26.27±9.67	<0.001*
Hataysal et al.,(2022) Turkey (99)	Visfatin	136.12(80.4-204.3) 25- 75%	66.58(40.4-88.9) 25-75%	<0.001*
Khan et al,.(2021) Pakistan (21)	IgA	41.89±14.82	33.53±11.59	0.018*
Ahmad et al.,(2021) India (88)	IgA α-amylase	2.98±1.66 68.42±26.28	5.62±1.77 83.53±27.61	0.001* 0.014*
Vasudevan et al.,(2021) India (94)	Carbonic anhydrase VI Total protein SOD	1925.54±1398.57 1.723±1.943 0.027±0.021	1444.17±1039.81 1.429±1.284 0.015±0.029	0.135 0.492 0.07
Al-Ali et al.,(2021) Iraq (104)	LL-37 Beta-defesin (HBD)	1.83±1.861 176.495±175.932	2.179±1.883 275.352±256.56	0.384 0.038*
Angarita-Diaz et al.,(2021) Colombia (108)	Statherin IgA LL-37	90,875.0 (IQR: 83,580.2-94,633.4) 37,776.42; IQR: 33,383.9-44,128.5 46.3; (IQR:40.1011-67.7)	94,734.6; (IQR: 92,934.6-95,113.7) 48,250.0; IQR: 31,461.9-67,418.8) 56.1; (IQR 43.6-116.2)	0.03* 0.12 0.56
Razi et al.,(2020) India (95)	IgA Total protein	85.0±14,3 32.8±1.2	106,3±28,5 28.9±1.1	0.015* 0.015*
Al-ani (2020) USA (107)	IgA APRP-1	207 (132.51) 422.96 (221.12)	237.55 (139.43) 389.15 (230.89)	0.388 0.566
Karpinska et al.,(2019) Poland (71)	MUC5B MUC7	0.38±0.32 1.39±0.86	0.63±0.35 5.47±1.18	0.0233* 0.0001*
Pyati et al.,(2018) India (78)	Total protein	0.41±0.15	0.34±0.12	0.017*
Nazemisalman et al.,(2018) Iran (102)	TNF-α	35.20 ± 16.23	26.20 ± 6.25	0.001*
Karpinska et al.,(2018) Poland (103)	APRP-1/2	34.45±4.85	15.70±3.05	0.001*
Sitaru et al., (2017) Romania (87)	α-Amylase	158.49±2.07	149,5±2.35	0.001*
Prester et al.,(2017) Croatia (72)	sCD14	Unstimualted 203.3±43.5 Stimulated 201±49.3	Unstimulated 167.9±33.1 Stimulated 105.7±26.8	<0.01*
Yang et al., (2015) China (105)	Proteinase-3	High caries group 11.07±7.10 Low caries group 12.79±6.19	17.82±7.31	0.009*
Hedenbjörck-Lager et al.,(2015) Sweden (106)	MMP-8 Total protein	Group II 345±371.3 Group III 426.7±355.4) Group II 826.2±403.5 Group III 1,010.6±629.4	254.6±189.8 794.4±397.2	<0.001* 0.011*
Cogulu et al.,(2015) Turkey (100)	IL-1B IL-1ra IL-10	74.64±48.57 227.1±111.93 3.86±4.57	66.69±39.02 240±106.94 1.77±1.29	>0.05 >0.05 >0.05
Hegde et al.,(2014) India (96)	Superoxidase mutase (SOD)	0.35±0.139	0.19±.0.049	<0.0001*
Golpasand Hagh et al., (2013) Iran (101)	IgA	6,020±760	12,320±19.9	0.009*
Pal et al., (2013) India. (97)	IgA	144.13± 20.85 (high caries) 189.13± 26.74 (low caries)	213.67± 28.67	< 0.05*

*Represent statistically significant difference <0.05.
